# Satellite-Based Spatiotemporal Trends in PM_2.5_ Concentrations: China, 2004–2013

**DOI:** 10.1289/ehp.1409481

**Published:** 2015-07-24

**Authors:** Zongwei Ma, Xuefei Hu, Andrew M. Sayer, Robert Levy, Qiang Zhang, Yingang Xue, Shilu Tong, Jun Bi, Lei Huang, Yang Liu

**Affiliations:** 1 State Key Laboratory of Pollution Control and Resource Reuse, School of the Environment, Nanjing University, Nanjing, Jiangsu, China; 2 Department of Environmental Health, Rollins School of Public Health, Emory University, Atlanta, Georgia, USA; 3 Goddard Earth Sciences Technology and Research, Universities Space Research Association, Greenbelt, Maryland, USA; 4 NASA Goddard Space Flight Center, Greenbelt, Maryland, USA; 5 Center for Earth System Science, Tsinghua University, Beijing, China; 6 Changzhou Environmental Monitoring Center, Changzhou, Jiangsu, China; 7 School of Public Health and Social Work and Institute of Health and Biomedical Innovation, Queensland University of Technology, Brisbane, Queensland, Australia

## Abstract

**Background:**

Three decades of rapid economic development is causing severe and widespread PM_2.5_ (particulate matter ≤ 2.5 μm) pollution in China. However, research on the health impacts of PM_2.5_ exposure has been hindered by limited historical PM_2.5_ concentration data.

**Objectives:**

We estimated ambient PM_2.5_ concentrations from 2004 to 2013 in China at 0.1° resolution using the most recent satellite data and evaluated model performance with available ground observations.

**Methods:**

We developed a two-stage spatial statistical model using the Moderate Resolution Imaging Spectroradiometer (MODIS) Collection 6 aerosol optical depth (AOD) and assimilated meteorology, land use data, and PM_2.5_ concentrations from China’s recently established ground monitoring network. An inverse variance weighting (IVW) approach was developed to combine MODIS Dark Target and Deep Blue AOD to optimize data coverage. We evaluated model-predicted PM_2.5_ concentrations from 2004 to early 2014 using ground observations.

**Results:**

The overall model cross-validation *R^2^* and relative prediction error were 0.79 and 35.6%, respectively. Validation beyond the model year (2013) indicated that it accurately predicted PM_2.5_ concentrations with little bias at the monthly (*R^2^* = 0.73, regression slope = 0.91) and seasonal (*R^2^* = 0.79, regression slope = 0.92) levels. Seasonal variations revealed that winter was the most polluted season and that summer was the cleanest season. Analysis of predicted PM_2.5_ levels showed a mean annual increase of 1.97 μg/m^3^ between 2004 and 2007 and a decrease of 0.46 μg/m^3^ between 2008 and 2013.

**Conclusions:**

Our satellite-driven model can provide reliable historical PM_2.5_ estimates in China at a resolution comparable to those used in epidemiologic studies on the health effects of long-term PM_2.5_ exposure in North America. This data source can potentially advance research on PM_2.5_ health effects in China.

**Citation:**

Ma Z, Hu X, Sayer AM, Levy R, Zhang Q, Xue Y, Tong S, Bi J, Huang L, Liu Y. 2016. Satellite-based spatiotemporal trends in PM_2.5_ concentrations: China, 2004–2013. Environ Health Perspect 124:184–192; http://dx.doi.org/10.1289/ehp.1409481

## Introduction

Fine particulate matter (PM_2.5_; particles with aerodynamic diameter ≤ 2.5 μm) has been strongly associated with adverse health effects (e.g., cardiovascular and respiratory morbidity and mortality) by numerous epidemiologic studies conducted primarily in developed countries (Pope and Dockery 2006). With the rapid economic development and urbanization occurring in China, severe, widespread PM_2.5_ pollution has attracted nationwide attention (Xu et al. 2013). However, research on the adverse health impacts of PM_2.5_ exposure has been hindered because a nationwide regulatory PM_2.5_ monitoring network did not exist until the end of 2012.

Estimating ground-level PM_2.5_ from satellite-retrieved aerosol optical depth (AOD) data is a promising, new method that has advanced rapidly in recent years (Hu et al. 2014b; Kloog et al. 2011; Lee et al. 2011; Liu et al. 2009). Satellite-driven statistical models have the potential to fill the spatiotemporal PM_2.5_ gaps left by ground monitors with high-quality predictions. Several recent studies of the health effects caused by long-term PM_2.5_ exposure have adopted satellite-estimated PM_2.5_ levels as their exposure estimates (Crouse et al. 2012; Madrigano et al. 2013). Because sufficient ground PM_2.5_ measurements are needed to fit and validate statistical models, development of models in China was difficult before 2013. van Donkelaar et al. (2010) estimated long-term (2001–2006) average global PM_2.5_ concentrations at 0.1° resolution using the PM_2.5_/AOD ratios derived from a global chemical transport model (CTM). Two follow-up studies estimated the global PM_2.5_ time series from 1998 to 2012 (Boys et al. 2014; van Donkelaar et al. 2015). Both studies validated their seasonal average estimates only with ground observations mostly obtained from North America, and the Pearson coefficients ranged from ~ 0.37 to ~ 0.68 (*R^2^* = ~ 0.14–0.46). Yao and Lu (2014) used an artificial neural network (ANN) model to estimate PM_2.5_ levels in China from 2006 to 2010. However, their ANN was trained partially using PM_2.5_ and satellite data from the United States, which may have introduced substantial prediction error.

Taking advantage of the newly available national PM_2.5_ measurements for China, Ma et al. (2014) estimated PM_2.5_ levels for 2013 in China using satellite AOD and a geographically weighted regression (GWR) model. Using an early version of the Dark Target (DT) algorithm (Remer et al. 2005), this study adopted a relatively coarse spatial resolution of 50 km but did not attempt to estimate historical PM_2.5_ levels. The coarse resolution was a result of the limited coverage of AOD values retrieved by the Moderate Resolution Imaging Spectroradiometer (MODIS; http://modis.gsfc.nasa.gov) instruments aboard the Terra and Aqua satellites launched by the National Aeronautics and Space Administration (NASA). In early 2014, more accurate Aqua MODIS Collection 6 (C6) AOD products retrieved by the enhanced DT (Levy et al. 2013) and Deep Blue (DB) algorithms (Hsu et al. 2013) were released. Despite providing better coverage over deserts and urban centers than DT AOD, DB AOD has rarely been used in PM_2.5_ studies owing to poorly characterized retrieval errors in earlier versions. As we demonstrate in the following sections, including the MODIS C6 DB AOD data substantially increases the spatiotemporal coverage of model predictions in China.

In this study, we developed a high-resolution (0.1°, which is approximately 10 km) statistical model to estimate historical ambient PM_2.5_ concentrations in China from 2004 to 2013 using MODIS C6 AOD data. First, we present our approach to generating a custom “combined” AOD parameter using the operational DT and DB AOD values, and we describe our two-stage spatial statistical model for estimating daily ambient PM_2.5_ levels. We then evaluate predicted PM_2.5_ concentrations at seasonal, monthly, and daily levels using ground PM_2.5_ measurements in China not included in the model development. Finally, we analyze the 10-year spatiotemporal trend of PM_2.5_ levels.

## Materials and Methods

**Figure 1:**
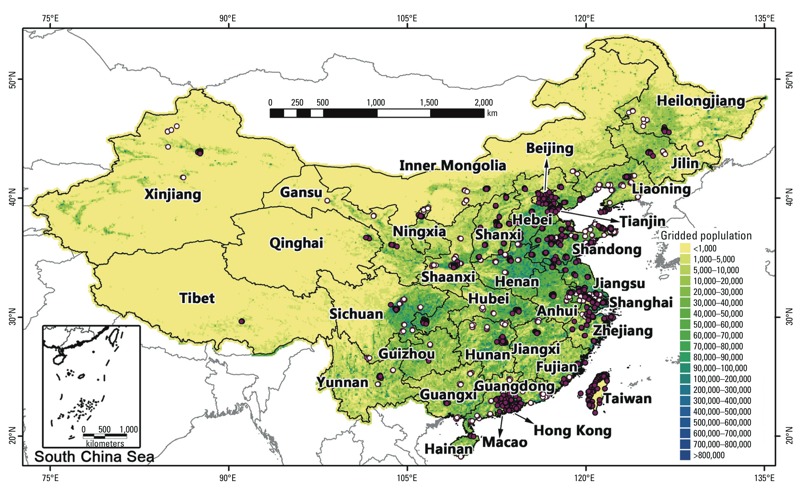
Spatial distribution of ground PM_2.5_ monitoring sites. Open circles denote the sites with data available only from January to June 2014. Solid circles denote the sites with data available for not only 2014 but also for 2013 or earlier years. Note that many clustered sites are overlapped because of their proximity. The spatial resolution of the background gridded population is 0.1° × 0.1°.

*Ground PM_2.5_ measurements.* The daily average PM_2.5_ concentrations for China (January 2013 to June 2014) were collected primarily from the website of the China Environmental Monitoring Center (CEMC). We collected additional data that are not included in the CEMC data from the websites of local environmental monitoring centers of several provinces (e.g., Shandong, Shanxi, Zhejiang, Guangdong) and municipalities (e.g., Beijing, Tianjin). Daily PM_2.5_ data for Macao (2013), Hong Kong (2005 to June 2014), and Taiwan (2004 to June 2014) were also collected from the websites of local environmental protection agencies. Data from the U.S. consulate sites in Beijing (2008–2013), Shanghai (2011–2013), Guangzhou (2011–2013), Shenyang (2013), and Chengdu (2012–2013) were also included. The web links for the abovementioned PM_2.5_ data sources are shown in Supplemental Material, Table S1. Data for Changzhou City in Jiangsu province were provided by the Changzhou Environmental Monitoring Center. Monthly and seasonal mean PM_2.5_ measurements for Beijing from 2005 to 2007 were obtained from Zhao et al. (2009). All ground PM_2.5_ measurements were obtained using tapered element oscillating microbalances (TEOMs) or beta attenuation monitors, both of which are subject to measurement errors due to the loss of semivolatile components (Duncan et al. 2014). However, because PM_2.5_ compliance in China is based on measurements obtained from these monitors, we used these PM_2.5_ measurements to develop and evaluate our model. Our study included a total of 1,185 monitoring sites in 205 cities or regions (Figure 1). The 2013 data were used for model fitting and cross-validation (CV), and data from other years were used to evaluate the predicted historical PM_2.5_ concentrations.

**Figure 2:**
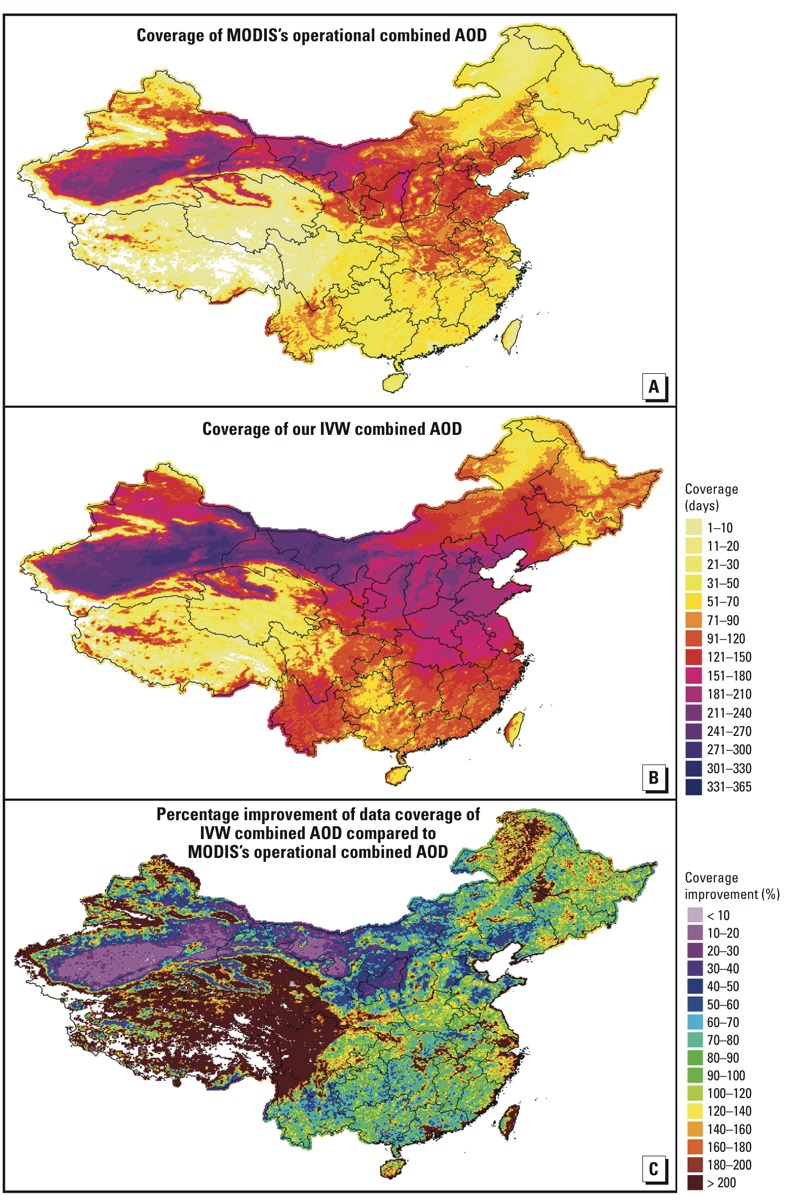
Spatial distribution of annual mean available days for MODIS’s operational combined AOD (*A*), our IVW–combined AOD (*B*), and percentage improvement of data coverage (*C*).

*Satellite data.* We extracted DT and DB AOD data for the period from January 2004 to June 2014 at 550 nm from the Aqua MODIS Level 2 aerosol data product, which were downloaded from the Level 1 and Atmospheric Archive and Distribution System (http://ladsweb.nascom.nasa.gov/). Aqua MODIS C6 includes an operational combined AOD product calculated from DB and DT AOD in three Normalized Difference Vegetation Index (NDVI) categories (Levy et al. 2013). This combined AOD is equal to DT AOD if NDVI > 0.3 and is equal to DB AOD if NDVI < 0.2. When 0.2 ≤ NDVI ≤ 0.3, the combined AOD equals the mean of DT and DB AOD if both values have high quality assurance (QA) flags. If one of the algorithms reports a higher QA than the other, then that AOD value is used. Detailed descriptions of the MODIS operational combined AOD algorithm and the QA flags can be found elsewhere (Levy et al. 2013). We did not use the operational combined AOD data set of the MODIS C6 aerosol product because it discarded all DB AOD data with NDVI values > 0.3 (see Supplemental Material, “Validation of Aqua MODIS C6 AOD products”). We developed a three-step customized approach to combine DT and DB AOD. First, we performed a regression analysis between the daily collocated DT and DB AOD. The resulting regression coefficients were then used to predict the missing DB AOD in those pixels with only DT AOD and vice versa (Puttaswamy et al. 2014). Second, Level 2–validated AOD observations from 33 Aerosol Robotic Network (AERONET) sites (see Supplemental Material, Figure S1) in China were matched with the gap-filled MODIS DT and DB AOD retrievals. The variance of the differences between gap-filled DT (or DB) AOD and AERONET AOD values for each season was calculated. Finally, we combined the gap-filled DT and DB AOD data using the inverse variance weighting (IVW) approach as follows:


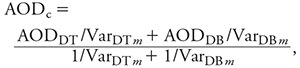
[1]

where AOD_c_ is the IVW–combined AOD; AOD_DT_ and AOD_DB_ are the gap-filled DT and DB AOD, respectively; and Var_DT_*_m_* and Var_DB_*_m_* are the variances of the differences between the gap-filled DT and DB AOD and the AERONET AOD of season *m*, respectively. When compared with the AERONET observations, our combined AOD performed similarly (*R^2^* = 0.80, mean bias = 0.07) to MODIS’s operational combined AOD (*R^2^* = 0.81, mean bias = 0.07) but had 90% greater coverage. Spatially, the improvement in temporal coverage varied by land use type (Figure 2). Coverage for densely populated southern and eastern China improved by 50–100%. The Tibetan plateau showed the most improvement (~ 200%), whereas the Gobi and Taklamakan Deserts showed the least (20–30%).

To account for the impact of fire smoke on PM_2.5_ levels (Hu et al. 2014c), we downloaded Aqua and Terra MODIS active fire spots from 2004 to 2014 from the NASA Fire Information for Resource Management System (https://earthdata.nasa.gov/data/near-real-time-data/firms).

*Meteorological and land use data.* Goddard Earth Observing System Data Assimilation System GEOS-5 Forward Processing (GEOS 5-FP) (Lucchesi 2013) and GEOS-5.2.0 meteorological data were used in this study. GEOS-5 FP is the latest version of GEOS-5 meteorological data, with a spatial resolution of 0.25° latitude × 0.3125° longitude in a nested grid covering China and has been available since April 2012. GEOS-5.2.0 is the previous version of GEOS-5 FP and has a resolution of 0.50° × 0.666°. GEOS-5.2.0 data are available from January 2004 to May 2013. We averaged GEOS-5 FP data to the GEOS-5.2.0 grid to maintain a consistent spatial resolution across all model years. We used GEOS-5 FP data from 2013 for model development and GEOS-5.2.0 data from 2004 to 2012 for estimating historical PM_2.5_ levels. The period of overlap (April 2012 to May 2013) for GEOS-5 FP and GEOS-5.2.0 data was used to evaluate the influence of the change in meteorological data source (see Supplemental Material, Figure S3, Table S2, “Comparison of model performance using GEOS-5 FP and GEOS-5.2.0 meteorological data”). We extracted planetary boundary layer height (PBLH, 100 m), wind speed (WS, meters per second) at 10 m above the ground, mean relative humidity in PBL (RH_PBLH, percent), and surface pressure (PS, hectopascals) between 1300 and 1400 hours local time (Aqua satellite overpass time corresponds to 1330 hours local time), as well as cumulative precipitation from the previous day (Precip_Lag1; millimeters). Land use variables at 300-m resolution were obtained from the European Space Agency (ESA) Global Land Cover data portal (GlobCover; http://due.esrin.esa.int/page_globcover.php) (Bontemps et al. 2011). We extracted urban and forest cover data from GlobCover 2005–2006 to represent study years 2004–2008 and from GlobCover 2009 to represent the years 2009–2014.

*Data integration.* We created a 0.1° grid (100,699 grid cells in total) for data integration and model development. Ground PM_2.5_ data from multiple monitors in each grid cell were averaged. Because the sizes and geographical locations of MODIS AOD pixels vary in space and time, a 0.1° grid cell may have multiple AOD pixels (e.g., near the center of each satellite swath), or an AOD pixel may cover multiple 0.1° grid cells (e.g., near the edge of each swath). Therefore, Thiessen polygons representing individual MODIS AOD pixels were created and then mapped to the 0.1° grid to spatially assign combined AOD values to the grid cells. We interpolated the GEOS-5 FP and GEOS-5.2.0 data to the 0.1° grid using the inverse distance weighting (IDW) method. We calculated the percentage forest cover and urban area in each grid cell and the daily total counts of MODIS fire spots for each grid cell using a 75-km radius buffer. Finally, all of the variables in 2013 were matched by grid cell and day-of-year (DOY) for model fitting. The model prediction data set was composed of all spatiotemporally matched variables except PM_2.5_ concentrations from January 2004 to June 2014. Before model development, the independent variables in the fitting and prediction data sets were centered by subtracting their respective mean values computed from the fitting data set.

*Model development and validation.* We developed a two-stage statistical model to calibrate the spatiotemporal relationships between PM_2.5_ and AOD. The first-stage linear mixed-effects (LME) model included day-specific random intercepts and slopes for AOD and season-specific random slopes for meteorological variables:

*PM*_2.5,_*_st_* = (μ + μ´) + (β_1_ + β_1_´)*AOD_st_* + (β_2_ + β_2_´)*WS_st_* + (β_3_ + β_3_´)*PBLH_st_* + (β_4_ + β_4_´)*PS_st_* + (β_5_ + β_5_´)*RH_PBLH_st_* + β_6_*Precip_Lag*1*_st_* +β_7_*Fire_spots_st_* + ε_1,_*_st_*(μ´, β_1_´) ~ N[(0,0), Ψ_1_] + ε_2,_*_sj_*(β_2_´, β_3_´, β_4_´, β_5_´) ~ N[(0,0,0,0), Ψ_2_], [2]

where *PM*_2.5,_*_st_* is the average observed PM_2.5_ concentration at grid cell *s* on DOY *t*; *AOD_st_* is IVW–combined AOD*; WS_st_*, *PBLH_st_*, *PS_st_*, *RH_PBLH_st_*, and *Precip_Lag*1*_st_* are meteorological variables; *Fire_spots_st_* is the fire count; μ and μ´ are the fixed and day-specific random intercepts, respectively; β_1_–β_7_ are fixed slopes for independent variables; β_1_´ is the day-specific random slope for AOD; β_2_´–β_5_´ are the season-specific random slopes for meteorological variables; ε_1,_*_st_* is the error term at grid cell *s* on day *t;* ε_2,_*_sj_* is the error term at grid cell *s* in season *j*; Ψ_1_ and Ψ_2_ are the variance–covariance matrices for the day- and season-specific random effects, respectively; and N represents normal distribution. In addition to modeling season-specific meteorological random effects, we tested alternative models with day- and month-specific random effects for meteorological variables and found that this may cause over-fitting (data not shown).

We fitted the first-stage model for each province separately. Because the provinces in western China (e.g., Tibet, Xinjiang, Qinghai) do not have enough PM_2.5_ monitoring sites (Figure 1) to produce a robust model-fitting data set, we created a buffer zone for each province to include at least 3,000 data records and at least 300 days in 2013. We averaged overlapping predictions from neighboring provinces to generate a smooth national PM_2.5_ concentration surface.

The second-stage generalized additive model (GAM) is expressed as follows:

*PM*_2.5_*_resid_st_* = μ_0_ + *s*(*X*, *Y*)*_s_* + *s*(*ForestCover*)*_s_* + *s*(*UrbanCover*)*_s_* + ε*_st_*, [3]

where *PM*_2.5_*_resid_st_* is the residual from the first-stage model at grid cell *s* on day *t*; μ_0_ is the intercept term; *s*(*X*, *Y*)*_s_* is the smooth term of the coordinates of the centroid of grid cell *s*; *s*(*ForestCover*)*_s_* and *s*(*UrbanCover*)*_s_* are the smooth functions of percent forest cover and urban area for grid cell *s*, respectively; and ε*_st_* is the error term.

Statistical indicators, such as the coefficient of determination (*R^2^*), mean prediction error (MPE), root mean squared prediction error (RMSE), and relative prediction error (RPE; defined as RMSE divided by the mean ground PM_2.5_), were calculated and compared between model fitting and cross-validation to assess model performance and to test for potential model over-fitting.

*Prediction, evaluation, and time-series analysis of historical PM_2.5_.* The historical daily PM_2.5_ concentrations (2004–2012) were estimated using the model developed based on 2013 data, assuming that the daily relationship between PM_2.5_ and AOD was constant for the same DOY in each year. Because there were few ground PM_2.5_ measurements for mainland China before 2013, we estimated daily PM_2.5_ concentrations in the first half of 2014 using the model established for 2013 and compared them with the ground measurements to validate the accuracy of the historical PM_2.5_ estimations. We evaluated historical PM_2.5_ predictions (including 2014) at daily, monthly, and seasonal scales. Because some AOD-derived PM_2.5_ estimates are missing owing to cloud and snow surfaces, we conducted a sensitivity analysis to test how many AOD-derived PM_2.5_ estimations could represent the true monthly and seasonal mean PM_2.5_ concentrations. We required each evaluation grid cell to have at least 25 PM_2.5_ ground measurements in a given month to calculate the monthly mean PM_2.5_ concentration and at least 25 measurements in each month of a season to calculate the seasonal mean PM_2.5_ concentration.

We calculated the monthly mean PM_2.5_ anomaly time series by subtracting the 10-year average PM_2.5_ concentration of the corresponding month for each grid cell and analyzed the PM_2.5_ trend for each grid cell using least squares regression (Weatherhead et al. 1998), which has been applied to global analyses of monthly mean AOD anomaly time-series data (Hsu et al. 2012). For each grid cell, we required at least six daily PM_2.5_ predictions in each month to calculate the monthly mean PM_2.5_ and at least 6 months of anomaly data per year to be included in the time-series analysis.

**Figure 3:**
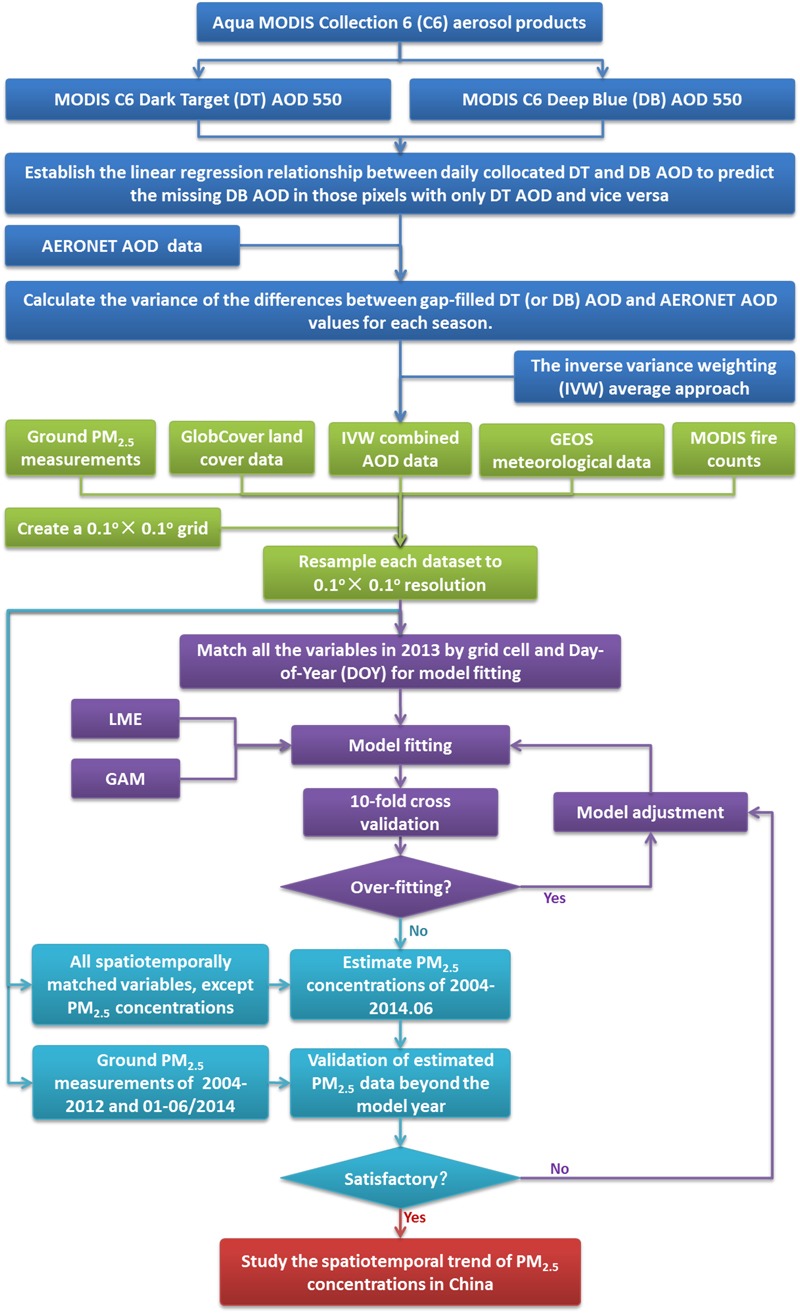
Workflow for estimating spatiotemporal PM_2.5_ concentrations.

The workflow of estimating the spatiotemporal PM_2.5_ concentrations in this study is shown in Figure 3.

## Results

*Descriptive statistics of the model-fitting data set.* A total of 63,031 data records were included in the final 2013 model-fitting data set. The overall mean PM_2.5_ concentration was 77.05 μg/m^3^, and the mean value of our combined AOD was 0.69 (see Supplemental Material, Table S3). These results are approximately five times higher than those obtained for the eastern and southeastern United States (Hu et al. 2013; Liu et al. 2005).

*Results of model fitting and cross-validation.* We summarize the fixed effects estimates, model fitting, and CV results of the first-stage LME model for each province in Supplemental Material, Table S4. AOD is the only variable that was statistically significant in all provincial models (*p* < 0.05). Wind speed, relative humidity, and precipitation were significant in most provincial models. Fire spots were not significant in some provinces, most likely because these regions have infrequent fire activity. The CV *R^2^* values of the first-stage LME model ranged from 0.64 in Ningxia to 0.82 in Zhejiang. The spatial distribution of first-stage LME CV *R^2^* (not shown here) indicates that our LME model generally performed better in south, east, north, and northeast China than in west and northwest China, which have fewer PM_2.5_ monitoring networks (Figure 1).

**Figure 4:**
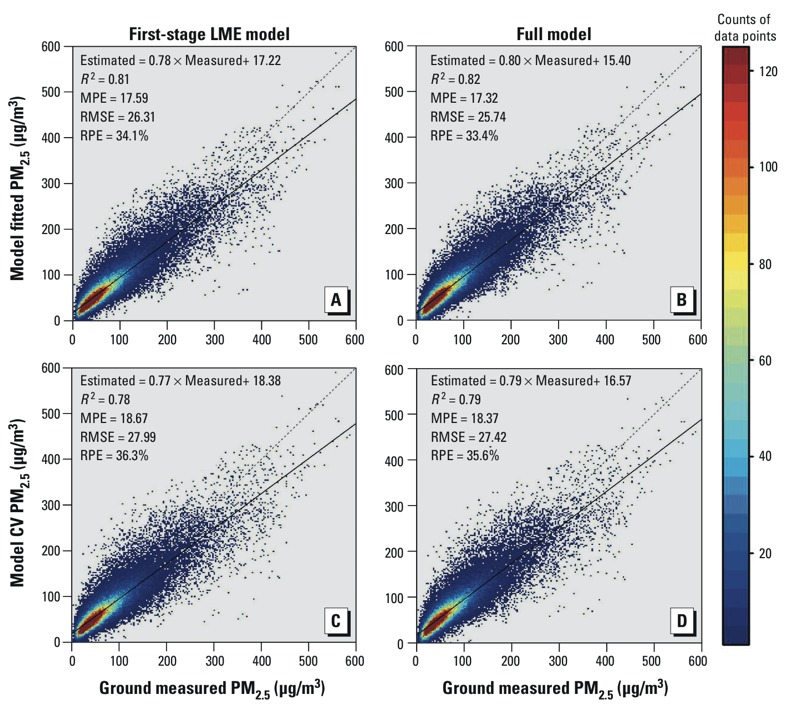
Density scatterplots of model fitting and cross-validation (CV) at the daily level (*n* = 63,031). (*A*) and (*B*) are model-fitting results for the first-stage linear mixed effects (LME) model and the full LME + generalized additive model (GAM) model, respectively. (*C*) and (*D*) are model CV results for the first-stage LME model and the full LME + GAM model, respectively. Abbreviations: MPE, mean prediction error (μg/m^3^); RMSE, root mean squared prediction error (μg/m^3^); RPE, relative prediction error (%). The dashed line is the 1:1 line.

Figure 4 shows the model-fitting and CV results for the first-stage and full models. The full-model fitting and CV *R^2^* values are 0.82 (Figure 4B) and 0.79 (Figure 4D), respectively, indicating that this model was not substantially over-fitted. Comparing the first-stage model (Figure 4A,C) with the full model (Figure 4B,D), it is clear that the second-stage GAM model marginally increased the *R^2^* values. However, the GAM model did increase the slope (from 0.77, Figure 4C, to 0.79, Figure 4D) and reduce the intercept (from 18.38, Figure 4C, to 16.57 μg/m^3^, Figure 4D) of the linear regression between the model-estimated and observed PM_2.5_ concentrations for 2013. More importantly, the PM_2.5_ levels in Hebei Province predicted by the full model were approximately 20 μg/m^3^ higher than those predicted by the first-stage model; the predicted PM_2.5_ levels in Tibet were approximately 15 μg/m^3^ lower in the full model (see Supplemental Material, Figure S4) than in the first-stage model, showing that the spatial pattern of the PM_2.5_ levels predicted by the full model was more consistent with that of the ground observations than PM_2.5_ levels predicted by the first-stage model.

**Figure 5:**
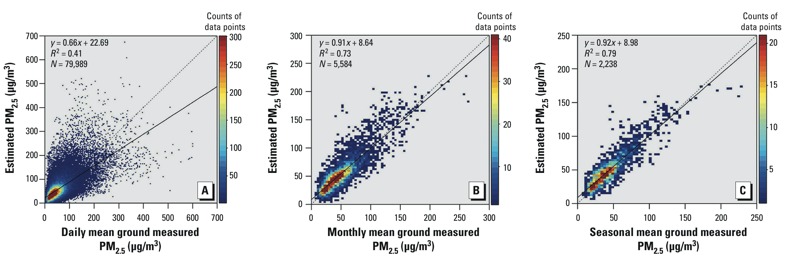
Evaluation of historical PM_2.5_ estimations (2004–2012 and January–June 2014) at daily (*A*), monthly (*B*), and seasonal (*C*) levels. Because there were few ground PM_2.5_ data for mainland China before 2013, we also estimated PM_2.5_ for the first half of 2014 using the 2013 model and compared the results with the ground measurements to validate the accuracy of the historical estimations.

*Evaluation of historical PM_2.5_ predictions.* Although our model’s predictions for daily level observations were poor compared with the historical observations (*R^2^* = 0.41, *n* = 79,989) (Figure 5A), it performed much better at the monthly and seasonal levels (Figure 5B and C, respectively). The sensitivity analysis showed that more daily predictions yielded more accurate monthly or seasonal estimations (see Supplemental Material, Figure S5). Figure 5B shows that the monthly mean satellite PM_2.5_ calculated from more than five predicted daily PM_2.5_ concentrations could be a fairly accurate (*R^2^* = 0.73) representation of monthly PM_2.5_ levels measured from ground observations with only a slight bias (regression slope = 0.91). This threshold of 6 days per month is consistent with the method of a previous global AOD trend study (Hsu et al. 2012). At the seasonal level (Figure 5C), satellite PM_2.5_ calculated from more than 10 predicted daily PM_2.5_ concentrations could be an accurate (*R^2^* = 0.79) representation of seasonal PM_2.5_ levels with little bias (regression slope = 0.92).

**Figure 6:**
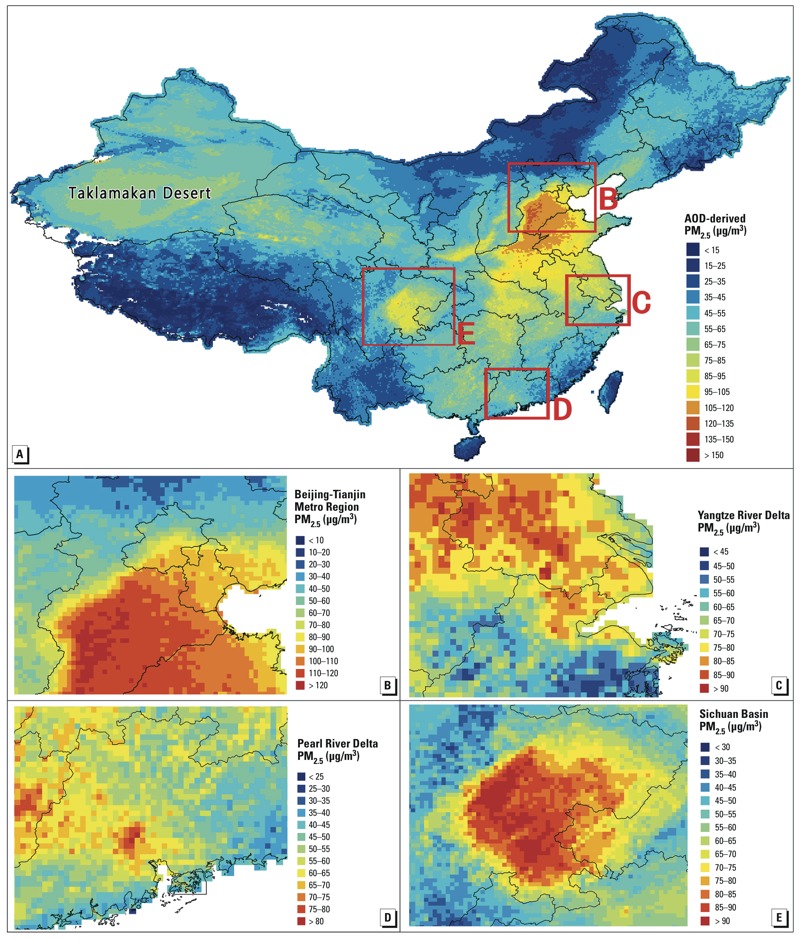
Spatial distributions of 10-year (2004–2013) mean PM_2.5_ estimations for all of China (*A*), the Beijing-Tianjin metropolitan region (*B*), the Yangtze River delta (*C*), the Pearl River delta (*D*), and the Sichuan Basin (*E*).

*Spatial and temporal PM_2.5_ concentration trends.* Figure 6 shows the spatial patterns of 10-year mean PM_2.5_ estimations (2004–2013) for China and four subregions (including the Beijing-Tianjin metropolitan region, the Yangtze River delta, the Pearl River delta, and the Sichuan Basin). The highest PM_2.5_ estimations were for the Beijing-Tianjin metropolitan region (including Beijing, Tianjin, and Hebei), followed by those for the Sichuan Basin, the Yangtze River delta (including Jiangsu, Shanghai, and Anhui), and the Pearl River delta. The 10-year mean PM_2.5_ estimations for the Beijing-Tianjin metropolitan region were generally ≥ 100 μg/m^3^, and the highest concentrations were ≥ 120 μg/m^3^. Similarly, the 10-year mean PM_2.5_ concentrations were generally ≥ 85 μg/m^3^ in the Sichuan Basin and the Yangtze River delta. The mean PM_2.5_ concentrations were generally ≥ 55 μg/m^3^ in the Pearl River delta. High PM_2.5_ levels also occured in the Taklamakan Desert in Xinjiang, an area that is a major dust source (Figure 6A). In the Supplemental Material, Figure S6 illustrates the seasonal patterns of the 10-year mean PM_2.5_ concentrations in China. Winter was the most polluted season (mean PM_2.5_: 72.24 μg/m^3^), and summer was the cleanest season (32.90 μg/m^3^).

**Figure 7:**
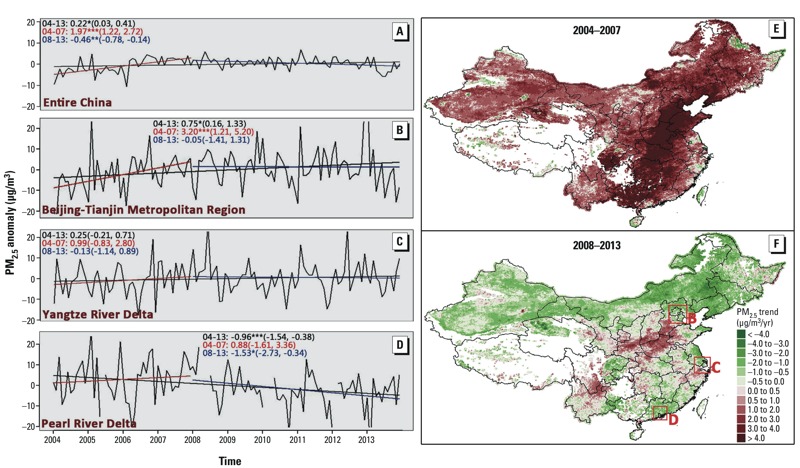
Time series of monthly, satellite-derived PM_2.5_ anomaly (μg/m^3^) for all of China (*A*), the Beijing-Tianjin metropolitan region (*B*), the Yangtze River delta (*C*), and the Pearl River delta (*D*); and spatial distribution of PM_2.5_ trends for 2004–2007 (*E*) and 2008–2013 (*F*). The white areas in (*E*) and (*F*) indicate missing data. The black lines in *A*–*D* denote the PM_2.5_ trends for 2004–2013, the red lines represent the trends for 2004–2007, and the blue lines represent the trends for 2008–2013. The PM_2.5_ trends (μg/m^3^ per year), 95% confidence intervals (CIs) in parentheses (μg/m^3^ per year), and significance levels (*p < 0.05; **p < 0.01; ***p < 0.005) are also shown in *A*–*D*.

Figure 7 shows that although China had an overall positive 10-year average PM_2.5_ trend of 0.22 μg/m^3^ per year (Figure 7A), there was significant regional variability. For example, the Beijing-Tianjin metropolitan region had more rapid increases in PM_2.5_ (0.75 μg/m^3^ per year) than the rest of the nation (Figure 7B), whereas the Pearl River delta experienced a rapid decrease (0.96 μg/m^3^ per year) (Figure 7D). The PM_2.5_ level in the Yangtze River delta region remained steady (Figure 7C). In addition, PM_2.5_ levels in most of China increased by 1.97 μg/m^3^ per year before 2008 but decreased by 0.46 μg/m^3^ per year afterwards (Figure 7A,E,F). Similar trends were observed in the Beijing-Tianjin metropolitan region (Figure 7B). The PM_2.5_ level remained relatively constant in the Pearl River delta from 2004–2007, followed by a negative trend of 1.53 μg/m^3^ per year after 2008 (Figure 7D).

## Discussion

Compared with our previous GWR model (CV *R^2^* = 0.64) (Ma et al. 2014), the two-stage model presented herein demonstrated superior performance (CV *R^2^* = 0.79). The CV RPE decreased from 51.3% (Ma et al. 2014) to 35.6% (the present study), approaching results seen in regional-scale studies conducted in the United States (Hu et al. 2014a; Lee et al. 2011). This improvement is particularly encouraging for our national model because, unlike regional-scale models, the PM_2.5_–AOD relationship will inevitably vary in space (e.g., variable PM_2.5_ composition and vertical distribution caused by different emission sources; variation of synoptic weather patterns by province). The first-stage CV *R^2^* dropped to 0.63 if a single LME model was fitted for the whole domain, further illustrating that a constant daily PM_2.5_–AOD relationship is a valid assumption only for relatively small geographic regions. Using both MODIS C6 DT and DB AOD to obtain a custom combined AOD yielded a 25-fold increase in spatial resolution (from 50 to 10 km) and greatly improved the AOD data coverage. There were 120% more matched DB AOD values than DT AOD values when comparing DB and DT AOD data with AERONET observations (see Supplemental Material, Figure S2). Furthermore, our analysis indicated that in China, DB AOD had a smaller mean bias overall than DT AOD (0.01 ~ 0.05 vs. 0.13 ~ 0.18) (see Supplemental Material, Figure S2), enabling us to estimate lower PM_2.5_ levels.

To our knowledge, this is the first national-scale study in China to use advanced statistical models to estimate and evaluate historical PM_2.5_ levels in the years beyond the modeling year. The lack of concordance between daily historical PM_2.5_ predictions and ground measurements was caused by the strong model assumption that the daily PM_2.5_–AOD relationship derived from the 2013 data remained constant for the same DOY in each year. This limitation of our model cannot be resolved without sufficient historical PM_2.5_ data to allow annual model adjustments before 2013. Nonetheless, our monthly (*R^2^* = 0.73, slope = 0.91) and seasonal (*R^2^* = 0.79, slope = 0.92) mean PM_2.5_ predictions are accurate representations of the ground measurements with relatively low biases and can serve as exposure estimates to study the health impacts of long-term PM_2.5_ exposure in China. The seasonal patterns showed that the most polluted season was winter and the cleanest was summer, consistent with the results of our previous study (Ma et al. 2014). Looking forward, this model can be fitted every year after 2013 to provide accurate daily PM_2.5_ concentrations and fill the spatial gaps left by the monitoring network.

Two approaches (including statistical and scaling models) can be applied to retrieve ground PM_2.5_ levels from satellite remotely sensed AOD data (Liu 2014). For statistical models to function properly, substantial ground data support is necessary. With the recently established ground monitoring network, we were able to develop this high-performance spatial model for China. The same model cannot be applied in regions with sparse or no ground observations. In this case, the scaling approach described by Brauer et al. (2012) is the only applicable method.

We compared the 9-year (2005–2013) AOD-derived and ground-measured PM_2.5_ trends for Hong Kong (no PM_2.5_ monitoring sites in 2004) and Taiwan (few sites in 2004). The results revealed that the AOD-derived PM_2.5_ trend for Hong Kong (–1.28 μg/m^3^ per year) was similar to the trend for the ground measurements (–1.35 μg/m^3^ per year). However, the AOD-derived PM_2.5_ trend for Taiwan was –0.17 μg/m^3^ per year, which was much higher than that for the ground measurements (–0.72 μg/m^3^ per year). This inconsistency is most likely due to missing satellite AOD retrievals. For example, only 34.5% of the grid cells in Taiwan had > 50% months with AOD-derived PM_2.5_ data. Missing AOD values are a major limitation of and challenge for PM_2.5_-AOD modeling (Liu 2014), and developing methods to account for missing AOD data in China will be a focus of our future research.

Nonetheless, the overall regional trends are consistent with the environmental policy and regulation change in China. We found an inflection point for the monthly mean PM_2.5_ time series around 2008. The PM_2.5_ level increased steadily between 2004 and 2007, but the trend reversed or became non-significant after 2008, especially in the Beijing-Tianjin metropolitan region. A recent study (Boys et al. 2014) also found that PM_2.5_ levels rose steadily until 2007 and then became stable in east Asia. China experienced a rapid growth of energy consumption before 2005 (Yuan et al. 2011), resulting in missed environmental quality targets between 2001 and 2005 (Xue et al. 2014). The growth in energy demand led to a stricter energy conservation and emissions reduction (ECER) policy, which required a 20% reduction in energy usage intensity by the end of 2010 compared with the level in 2005 (Lo and Wang 2013). The ECER policy was implemented in late 2006, and the overall reduction achieved by 2010 was 19.06% (Lo and Wang 2013). A recent study also showed that the production-related PM_2.5_ emissions in China peaked in approximately 2007 and dropped quickly afterwards (Guan et al. 2014). A sharp reduction of PM_2.5_ levels induced by this ECER policy may explain the inflection point.

## Conclusions

The two-stage satellite AOD model developed in the present study generated reliable historical monthly and seasonal PM_2.5_ predictions for China at 10-km resolution and with little bias, including data from the past decade, when the regulatory PM_2.5_ monitoring network did not exist. Because several long-term PM_2.5_ health effects studies in North America and the Global Burden of Disease project are driven by satellite exposure estimates obtained at this resolution (Brauer et al. 2012; Crouse et al. 2012; Madrigano et al. 2013), our model predictions could greatly enhance research on long-term PM_2.5_ health effects in China. With the release of the Terra MODIS C6 product in early 2015, the predicted historical PM_2.5_ time series can now be extended to early 2000, if consistent meteorological and land use parameters are found to cover 2000–2003. From 2013 onward, our model can provide daily PM_2.5_ exposure estimates to fill the gaps left by the PM_2.5_ monitoring network in China. Finally, given the wider dynamic range of PM_2.5_ concentrations in China compared with that in North America, likely due to intensive local sources, it is possible to further improve the performance of our model with detailed land use (e.g., road network) and emissions (e.g., major point sources) information, which was not available when this study was performed (Kloog et al. 2014).

## Supplemental Material

(2.2 MB) PDF
