# Supplementary material for: Satellite-Based Spatiotemporal Trends in PM2.5 Concentrations: China, 2004–2013
Source: Environ Health Perspect. 2015 Jul 24;124(2):184–92. doi: 10.1289/ehp.1409481 (PMC4749081; doi:10.1289/ehp.1409481)
Supplement: (2.2 MB) PDF [file ehp.1409481.s001.acco.pdf]

Environ Health Perspect
DOI: 10.1289/ehp.1409481

Note to Readers: *EHP* strives to ensure that all journal content is accessible to all readers. However, some figures and Supplemental Material published in *EHP* articles may not conform to 508 standards due to the complexity of the information being presented. If you need assistance accessing journal content, please contact ehp508@niehs.nih.gov. Our staff will work with you to assess and meet your accessibility needs within 3 working days.

Supplemental Material

Satellite-Based Spatiotemporal Trends in $PM_{2.5}$ Concentrations: China, 2004–2013

Zongwei Ma, Xuefei Hu, Andrew M. Sayer, Robert Levy, Qiang Zhang, Yingang Xue, Shilu Tong, Jun Bi, Lei Huang, and Yang Liu

Table of Contents

Table S1. The website links of $PM_{2.5}$ data sources

Validation of Aqua MODIS C6 AOD products

Figure S1. AERONET sites included in this study.

Figure S2. Validation of Aqua MODIS C6 DB and DT AOD products with different NDVI categories. The solid lines are the regression lines. The dot lines are the 1:1 lines. The dash lines indicate the expected error (EE) envelopes (± (0.05 + 20% of the AERONET AOD)) and the double EE envelopes (± 2 × (0.05 + 20% of the AERONET AOD)). Mean bias is defined as the mean value of the differences between AERONET AOD and satellite AOD values.

Comparison of model performance using GEOS-5 FP and GEOS-5.2.0 meteorological data

Figure S3. Comparison of CV estimations using GEOS-5.2.0 and GEOS-5 FP for the first-stage model (*A*) and the overall model (*B*) for Jan-May, 2013 (N=21,678)

Table S2. Summary statistics of daily comparisons between the estimations using GEOS-5.2.0 and GEOS-5 FP data (04/2012-12/2012, 273 days)

Table S3. Descriptive statistics of variables for the modeling dataset of 2013 (N=63,031)

Table S4. Fixed effect, model fitting and CV results of the first-stage LME model for each province

Figure S4. Annual mean $PM_{2.5}$ concentrations of 2013 for first-stage LME model (*A*), full model (*B*), and ground measurements (*C*). The mean ground-measured $PM_{2.5}$ concentrations are calculated from the days when corresponding gridded AOD values are available.

Figure S5. Sensitivity analysis of how many available AOD-derived $PM_{2.5}$ estimations in a month (A) or season (B) can represent a true monthly or seasonal mean value, respectively. Since there are fewer data points at the seasonal level, we use a 5-day moving time window here. For example, the label of "1-5" of X-axis in (B) means it includes available days from 1 to 5.

Figure S6. The spatial distributions of 10-year seasonal mean $PM_{2.5}$ estimations (2004-2013).

References

Table S1. The website links of $PM_{2.5}$ data sources

Data source	Website link
China Environmental Monitoring Center	http://113.108.142.147:20035/emcpublish/
Shandong	http://60.208.91.116:8801/AirDeploy.Web/Default.aspx?i=1
Shanxi	http://202.97.152.195:85/sx/
Zhejiang	http://115.236.164.226:8099/aqi/flex/index.html
Guangdong	http://www-app.gdepb.gov.cn/EQPubPlatform/
Beijing	http://zx.bjmemc.com.cn/
Tianjin	http://air.tjemc.org.cn/
Macao	http://www.smg.gov.mo
Hong Kong	http://www.aqhi.gov.hk/en.html
Taiwan	http://taqm.epa.gov.tw
U.S. consulate sites	http://www.stateair.net/web/mission/1/

Validation of Aqua MODIS C6 AOD products

We first validated the MODIS C6 DT and DB AOD products based on the NDVI categories defined by Levy et al. (2013). We processed MODIS 1 km monthly mean NDVI using data from LAADS Web (http://ladsweb.nascom.nasa.gov/). NDVI data within 3 km of the centroids of MODIS AOD pixels were averaged and matched with the AOD data. Aerosol Robotic Network (AERONET) Level 2 AOD data from the 33 sites (Figure S1) in China were downloaded from AERONET website (http://aeronet.gsfc.nasa.gov/). We interpolated AERONET AOD at 550 nm from AOD at 440 nm and 675 nm using the Angstrom Exponent. MODIS DT and DB AOD values within 6 km of the AERONET sites were directly matched with the AERONET sites. AERONET data within 30 minutes of the MODIS overpass time were extracted and averaged to compare with the DT and DB AOD data.

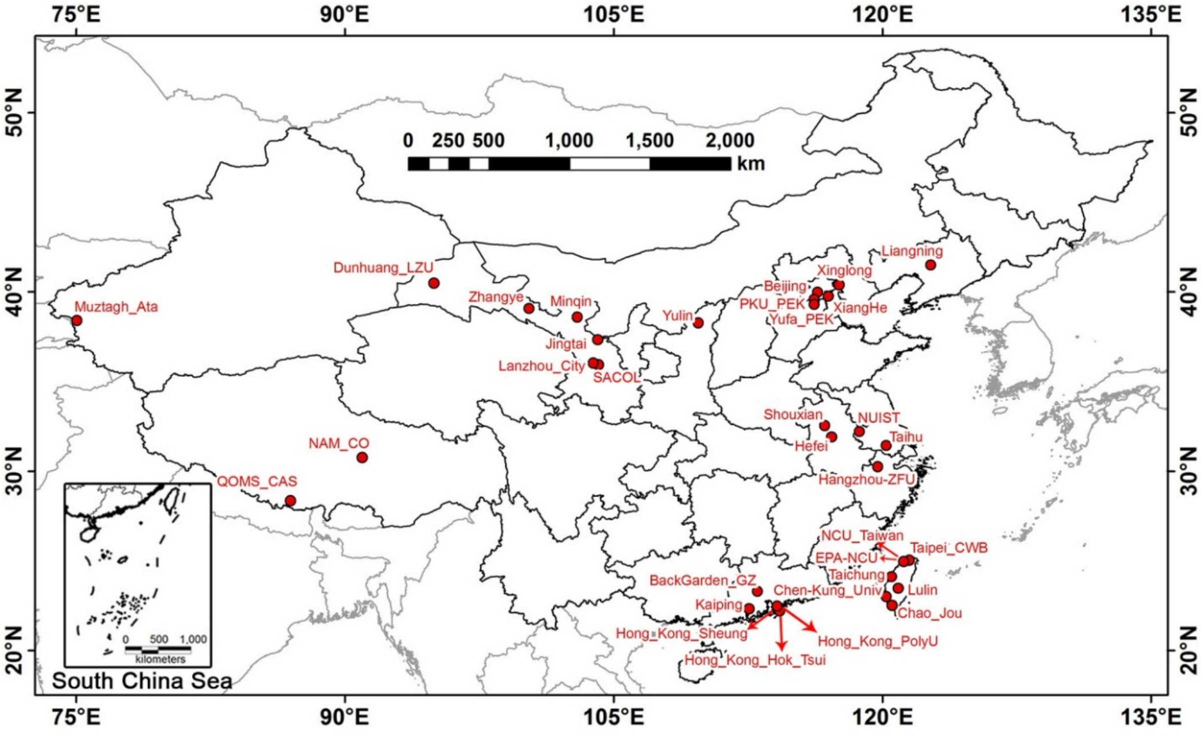

Figure S1. AERONET sites included in this study.

Figure S2 shows that DB AOD has more valid AOD retrievals and performs better than DT AOD in three NDVI categories. The fractions in expected error (EE) envelope (Sayer et al. 2013) of DB AOD are all higher than DT AOD. The regression lines of DB AOD are much closer to the 1:1 lines, indicating lower bias for DB AOD, which is also reflected by the low mean bias value of DB AOD. Our results show that the combination method introduced by Levy et al. (2013) has missed many AOD retrievals, especially in the category of NDVI>0.3 where DB AOD still performs well.

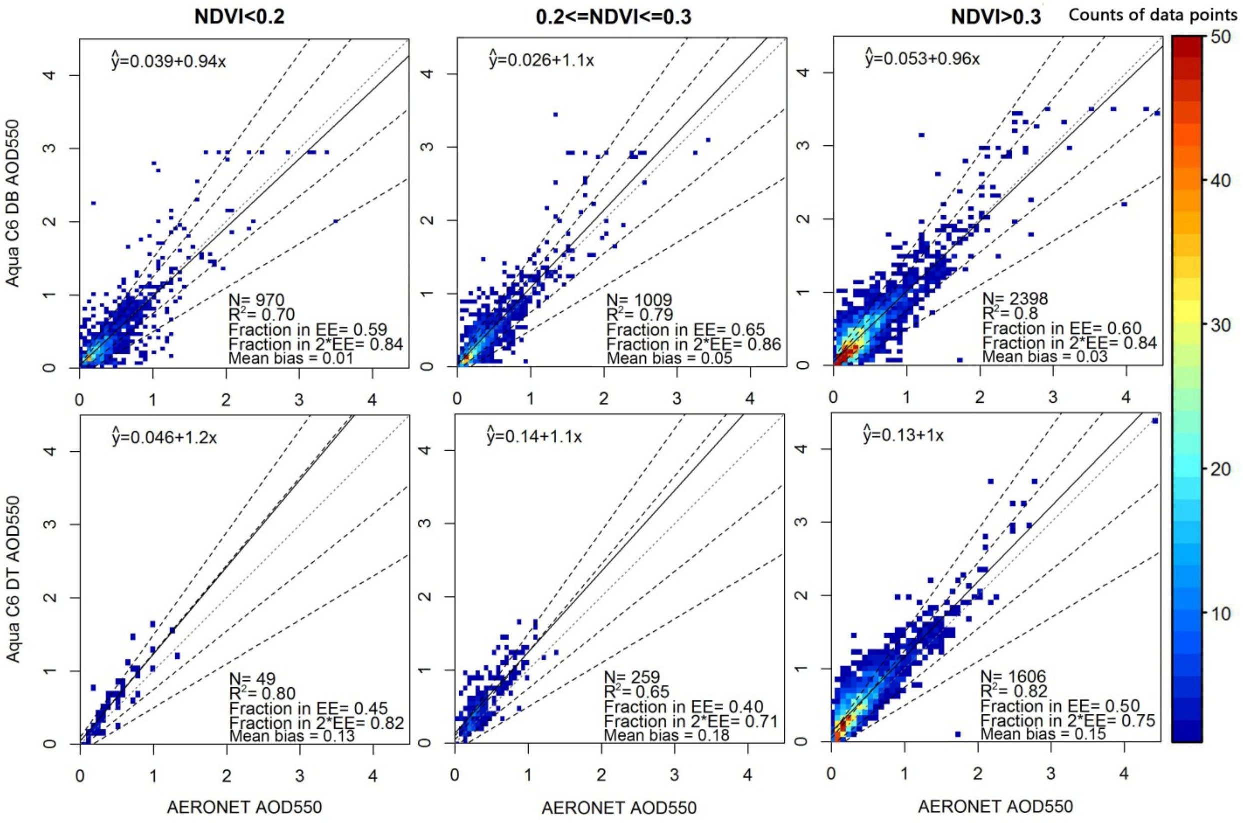

Figure S2. Validation of Aqua MODIS C6 DB and DT AOD products with different NDVI categories. The solid lines are the regression lines. The dot lines are the 1:1 lines. The dash lines indicate the expected error (EE) envelopes (± (0.05 + 20% of the AERONET AOD)) and the double EE envelopes (± 2 × (0.05 + 20% of the AERONET AOD)). Mean bias is defined as the mean value of the differences between AERONET AOD and satellite AOD values.

Comparison of model performance using GEOS-5 FP and GEOS-5.2.0 meteorological data

Since GEOS-5 FP and GEOS-5.2.0 data are both available from Apr, 2012 to May, 2013, we used the two versions GEOS-5 data in the overlaid period to evaluate the feasibility of using GEOS-5 FP data from 2013 for model fitting and using GEOS-5.2.0 data from 2004 to 2012 for historical $PM_{2.5}$ hindcasting. We performed the model CV again using the GEOS-5.2.0 data instead of GEOS-5 FP for 2013 and compared the CV results with those using GEOS-5 FP. The results show that the CV estimated $PM_{2.5}$ concentrations using GEOS-5.2.0 data are almost the same as the CV estimations using the GEOS-5 FP (Figure S3).

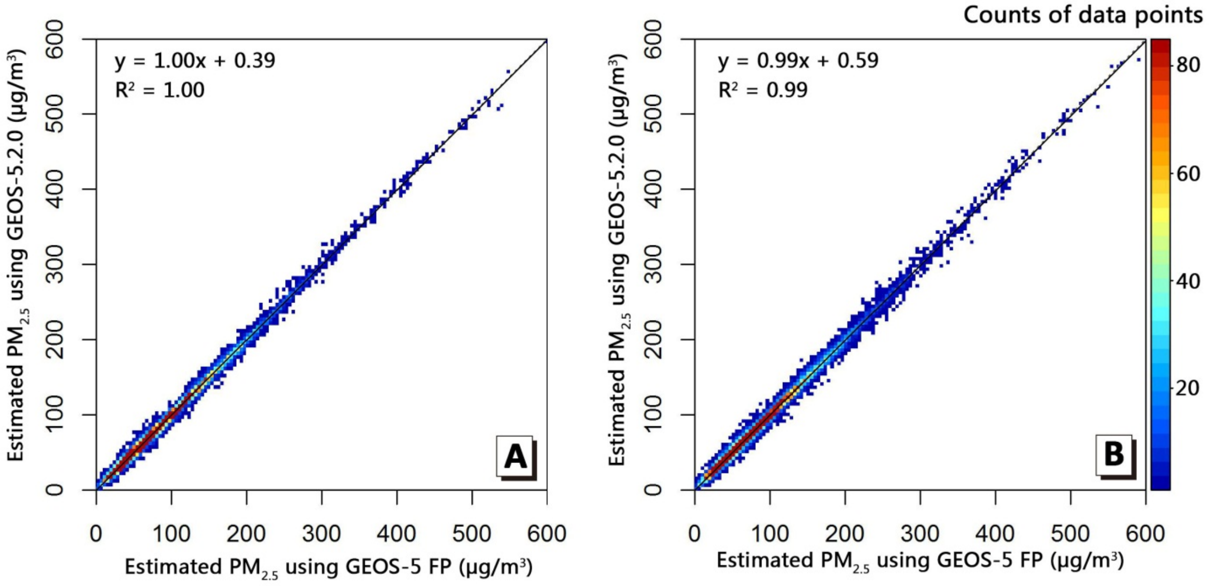

Figure S3. Comparison of CV estimations using GEOS-5.2.0 and GEOS-5 FP for the first-stage model (*A*) and the overall model (*B*) for Jan-May, 2013 (N=21,678)

We then estimated the daily gridded $PM_{2.5}$ concentrations for Apr-Dec, 2012 using the final model and the GEOS-5 FP data. We compared them with the corresponding estimations from the GEOS-5.2.0 data day by day. The results are shown in Table S2. The mean R^2 value, slope, and

intercept for the first-stage model are 0.90, 0.97, and -1.63, respectively. The mean R^2 value, slope, and intercept for the overall model are 0.92, 0.98, and -2.12, respectively, showing good consistency between historical estimations using GEOS-5.2.0 and GEOS-5 FP data. The daily R^2 values between estimations using GEOS-5.2.0 and GEOS-5 FP data range from 0.66 to 1.00 for the overall model. Only 7, 15, and 29 out of 273 days have the R^2 values less than 0.70, 0.80, and 0.85 respectively. The results show that it is feasible to use GEOS-5 FP data of 2013 for model fitting and validation and GEOS-5.2.0 data of 2004 to 2012 to estimate the historical $PM_{2.5}$ concentrations. Comparing the results of the first-stage and overall model, our second-stage GAM model can also reduce the uncertainties induced by the different versions of GEOS-5 datasets.

Table S2. Summary statistics of daily comparisons between the estimations using GEOS-5.2.0 and GEOS-5 FP data (04/2012-12/2012, 273 days)

	First-stage LME model			Overall model		
	R^2	Slope	Intercept	R^2	Slope	Intercept
Mean	0.90	0.97	-1.63	0.92	0.98	-2.12
median	0.92	0.98	-1.79	0.94	0.99	-2.05
10% percentile	0.80	0.88	-5.85	0.85	0.90	-5.93
5% percentile	0.72	0.83	-7.38	0.79	0.86	-7.49
Min	0.51	0.62	-16.2	0.66	0.72	-16.7
Max	1.00	1.17	13.4	1.00	1.18	8.47
STD	0.09	0.07	3.75	0.06	0.06	3.13

Table S3. Descriptive statistics of variables for the modeling dataset of 2013 (N=63,031)

Variables [a]	Min	Max	Median	Mean	S.D.
$PM_{2.5}$ (ug/m^3)	3.00	745.00	60.80	77.05	59.83
AOD (unitless)	-0.03	4.38	0.53	0.69	0.60
WS(m/s)	0.18	18.02	3.95	4.30	2.15
PBLH(100m)	1.37	51.89	15.55	16.24	5.18
PS (hPa)	561.8	1042.6	1001.6	978.3	58.9
RH_PBLH (%)	8.83	96.41	48.58	49.90	17.92
Precip_Lag1 (mm)	0.00	154.19	0.01	1.39	5.13
Fire_spots (counts)	0	366	0	2	9
ForestCover (%)	0.00	73.92	0.46	6.33	12.59
UrbanCover (%)	0.00	99.85	17.21	24.71	23.81

[a] Abbreviations used for the meteorological variables: WS: wind speed at 10 m above ground; PBLH: planetary boundary layer height; PS: surface pressure; RH_PBLH: mean relative humidity in planetary boundary layer; Precip_Lag1: cumulative precipitation of the previous day

DOI: 10.1289/ehp.1409481

Table S4. Fixed effect, model fitting and CV results of the first-stage LME model for each province

Province	N	Intercept [a]	Slope [a]							Fitting	CV
			AOD	WS [f]	PBLH	PS	RH_PBLH	Precip_Lag1	Fire_spots	R^2	R^2
Anhui	9477	80.51	40.56	-1.83			-94.35	-0.18	0.10	0.80	0.77
Chongqing	3484	86.00	42.10				-81.29			0.84	0.79
Fujian [b]	9812	60.07	32.46	-2.39		0.15	-55.59	-0.08		0.79	0.76
Gansu	4478	74.77	51.11	-3.98	-0.79			-0.63		0.77	0.70
Guangdong [c]	8471	61.59	31.02	-2.31		0.09	-59.88	-0.23	0.71	0.78	0.75
Guangxi	7123	62.54	37.80				-49.67	-0.20	0.65	0.79	0.75
Guizhou	4114	77.68	32.47			0.11	-50.67	-0.66		0.83	0.78
Hebei [d]	15030	82.62	56.11	-2.82		0.10	-85.57			0.77	0.75
Heilongjiang	13668	68.63	47.77	-1.17		0.14	-79.85	-0.15	0.40	0.80	0.78
Henan	10660	86.91	47.10				-73.08		0.15	0.74	0.70
Hubei	6748	83.30	42.81				-67.28	-0.43	0.15	0.79	0.75
Hunan	3688	80.42	45.63				-71.25	-0.30		0.85	0.78
Inner Mongolia	17387	79.50	57.73	-3.51			-56.86	-0.18	0.26	0.74	0.71
Jiangsu [e]	10855	103.92	40.13	-3.24		-0.72	-102.64	-0.21	0.09	0.79	0.76
Jiangxi	10980	65.92	40.81	-2.94		0.25	-88.42	-0.13	0.08	0.78	0.75
Jilin	15023	68.19	48.43			0.13	-78.68	-0.13	0.33	0.79	0.77
Liaoning	11536	64.85	43.77			0.27	-76.38	-0.14	0.34	0.81	0.78
Ningxia	6317	77.87	49.08	-2.94						0.70	0.64
Qinghai	4234	69.05	50.39	-3.92	-1.00		-41.72	-0.68		0.79	0.72
Shaanxi	5919	98.10	41.43			0.25				0.72	0.66
Shandong	14046	93.94	48.83			-0.53	-113.07		0.15	0.75	0.72
Shanxi	13064	84.35	55.95			0.07	-59.39			0.76	0.73
Sichuang	5135	75.28	46.57	-3.55	-0.78		-69.68	-0.60		0.77	0.72
Tibet	7172	73.81	44.63	-3.77	-1.43		-50.54	-0.89		0.73	0.67
Xinjiang	6311	78.86	49.09	-3.07	-1.11			-0.54		0.76	0.71
Yunnan	3302	70.62	37.54	-2.21			-76.44	-0.70		0.77	0.70
Zhejiang	9126	66.01	34.08	-2.19		0.21	-74.15			0.84	0.82

[a] Only statistically significant (p<0.05) intercepts and slopes are shown. [b] Including Taiwan. [c] Including Hong Kong, Macao, and Hainan. [d] Including Beijing and Tianjin. [e] Including Shanghai. [f] Abbreviations used for the meteorological variables: WS: wind speed at 10 m above ground; PBLH: planetary boundary layer height; PS: surface pressure; RH_PBLH: mean relative humidity in planetary boundary layer; Precip_Lag1: cumulative precipitation of the previous day.

DOI: 10.1289/ehp.1409481

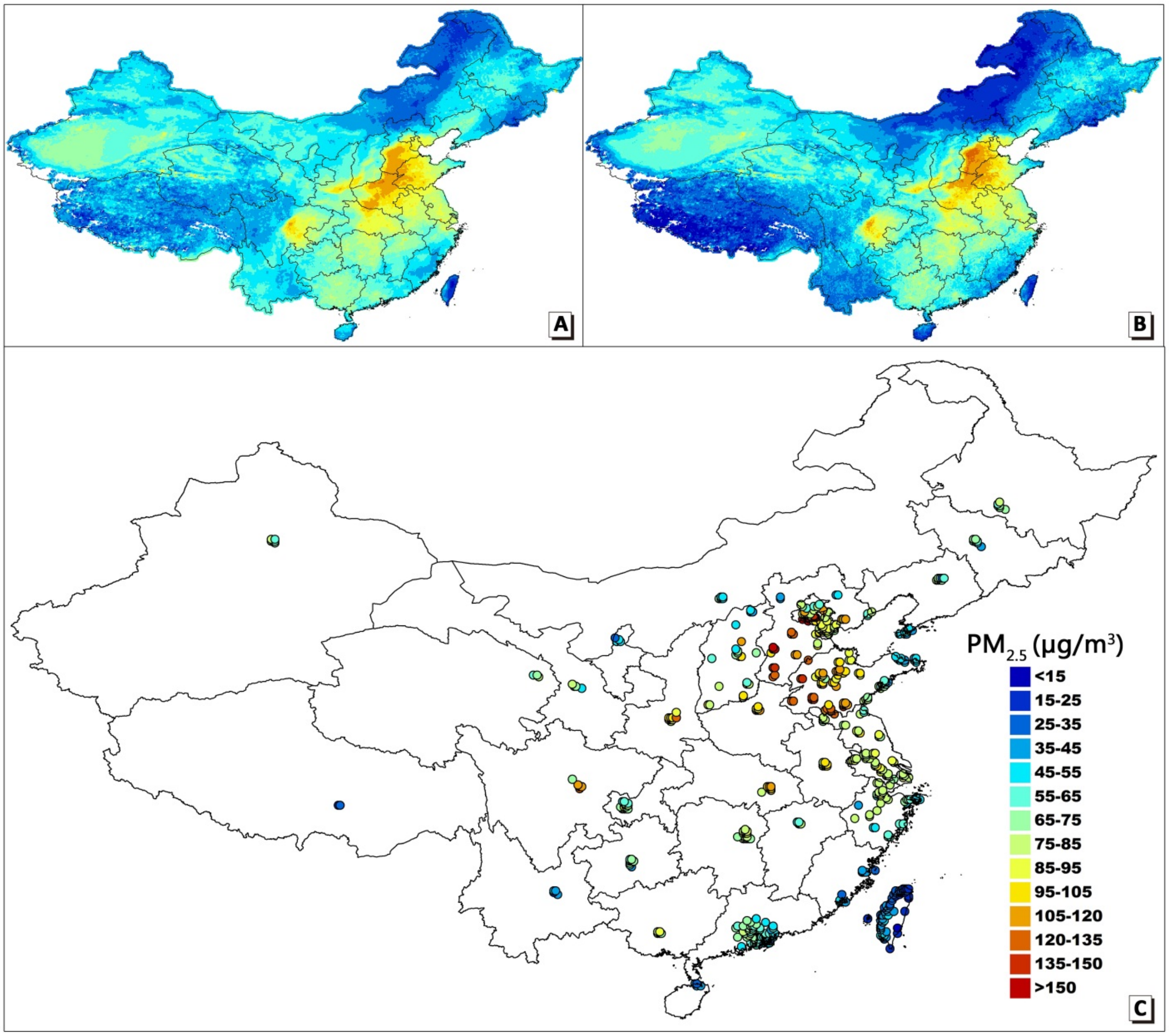

Figure S4. Annual mean $PM_{2.5}$ concentrations of 2013 for first-stage LME model (*A*), full model (*B*), and ground measurements (*C*). The mean ground-measured $PM_{2.5}$ concentrations are calculated from the days when corresponding gridded AOD values are available.

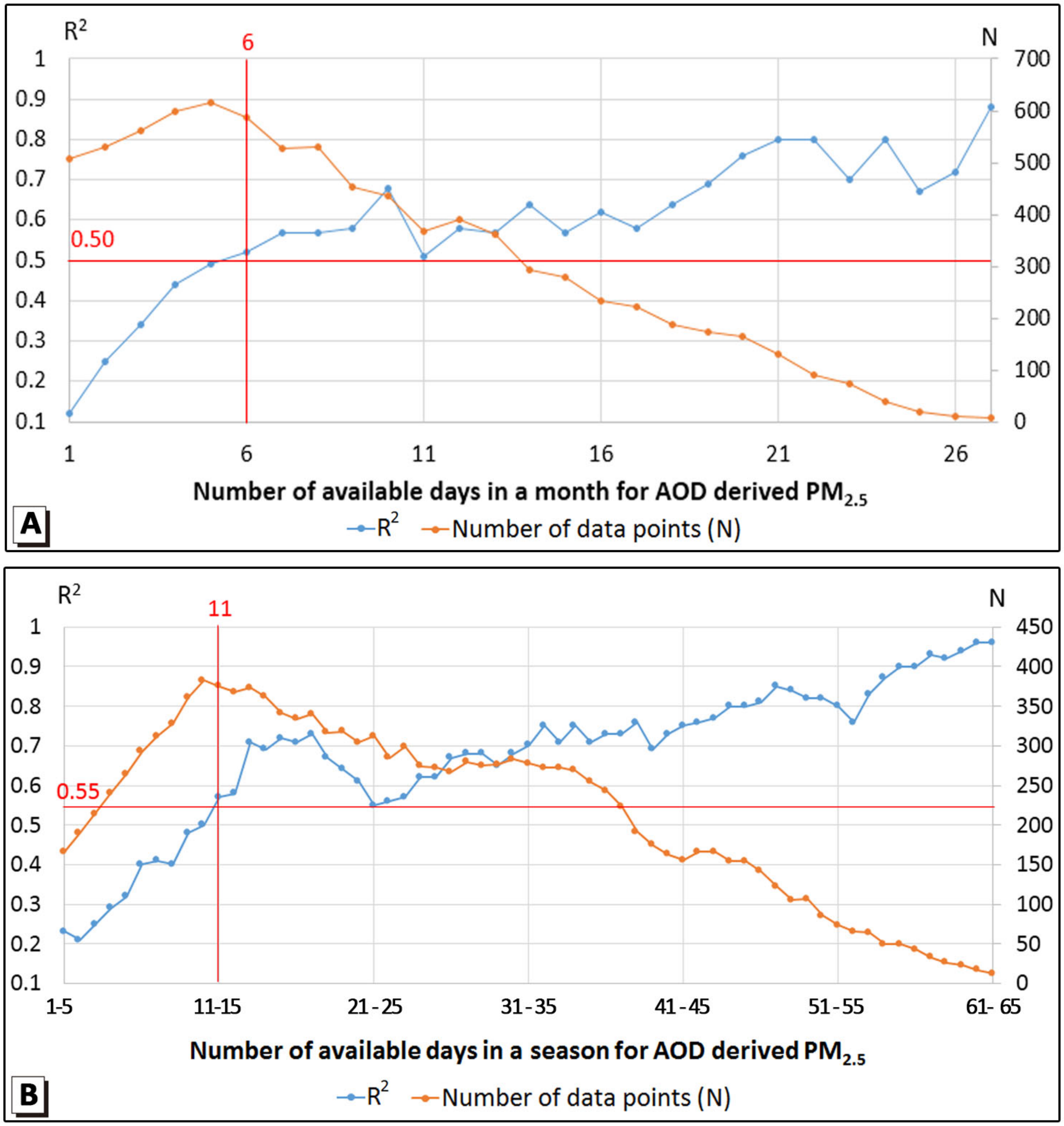

Figure S5. Sensitivity analysis of how many available AOD-derived $PM_{2.5}$ estimations in a month (A) or season (B) can represent a true monthly or seasonal mean value, respectively. Since there are fewer data points at the seasonal level, we use a 5-day moving time window here. For example, the label of "1-5" of X-axis in (B) means it includes available days from 1 to 5.

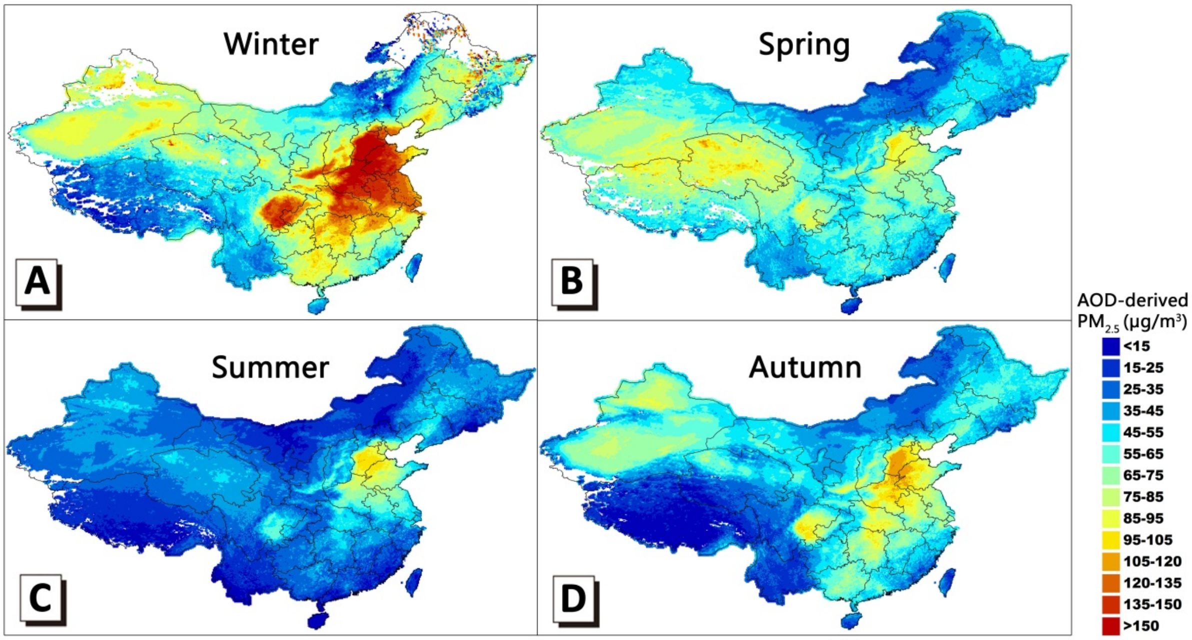

Figure S6. The spatial distributions of 10-year seasonal mean $PM_{2.5}$ estimations (2004-2013).

References

Levy R, Mattoo S, Munchak L, Remer L, Sayer A, Patadia F, et al. 2013. The Collection 6 MODIS aerosol products over land and ocean. Atmos Meas Tech 6:2989-3034.

Sayer AM, Hsu NC, Bettenhausen C, Jeong MJ. 2013. Validation and uncertainty estimates for MODIS Collection 6 “Deep Blue” aerosol data. J Geophys Res Atmos 118:7864-7872.
